# Crystal structures and electronic structures of SrSiSe_3_ and BaGeSe_3_

**DOI:** 10.1039/d6ra08053g

**Published:** 2026-09-29

**Authors:** Tristan Licskai, Abdeljalil Assoud, Ehsan Niknam, Holger Kleinke

**Affiliations:** a Department of Chemistry and Waterloo Institute for Nanotechnology, University of Waterloo Waterloo Ontario N2L 3G1 Canada kleinke@uwaterloo.ca

## Abstract

The structural diversity of ternary chalcogenides with the ABQ_3_ stoichiometry remains incompletely understood, particularly in systems where computational predictions have proposed structures that differ from experimentally accessible phases. This study reports the experimental crystal structures of the selenides SrSiSe_3_ and BaGeSe_3_, with the former being reported for the first time, and the latter revealing a unique structure type that differs from the previously calculated one. SrSiSe_3_ adopts the centrosymmetric monoclinic space group *P*2_1_/*c* with lattice parameters *a* = 6.6948(2) Å, *b* = 17.4606(4) Å, *c* = 15.8647(3) Å, *β* = 117.0100(10)°, and features pairs of edge-sharing SiSe_4_ tetrahedra. BaGeSe_3_ adopts the same space group, *P*2_1_/*c*, with *a* = 15.6603(7) Å, *b* = 6.0379(3) Å, *c* = 14.0072(6) Å, and *β* = 116.3830(10)°, but exhibits a distinctly different structural motif consisting of chains of corner-sharing GeSe_4_ tetrahedra. Comparisons with reported and calculated structures across the ABQ_3_ family highlight the substantial structural diversity accessible within this simple stoichiometry and demonstrate the importance of experimental structure determinations or global structure predictions in evaluating unknown structures. DFT calculations further indicate that SrSiSe_3_ and BaGeSe_3_ are semiconductors with calculated band gaps of 2.15 eV and 1.65 eV, respectively, placing both materials within the visible energy range. These results expand the known structural chemistry of ABQ_3_ ternary selenides and provide experimentally validated structures for assessing structure–property relationships and the reliability of computational predictions in this family.

## Abstract 2

Experimental discovery of monoclinic SrSiSe_3_ and structural reassessment of BaGeSe_3_ reveal distinct edge- and corner-sharing tetrahedral motifs, highlighting the crucial need for experimental synthesis over pure DFT predictions.

## Graphical abstract

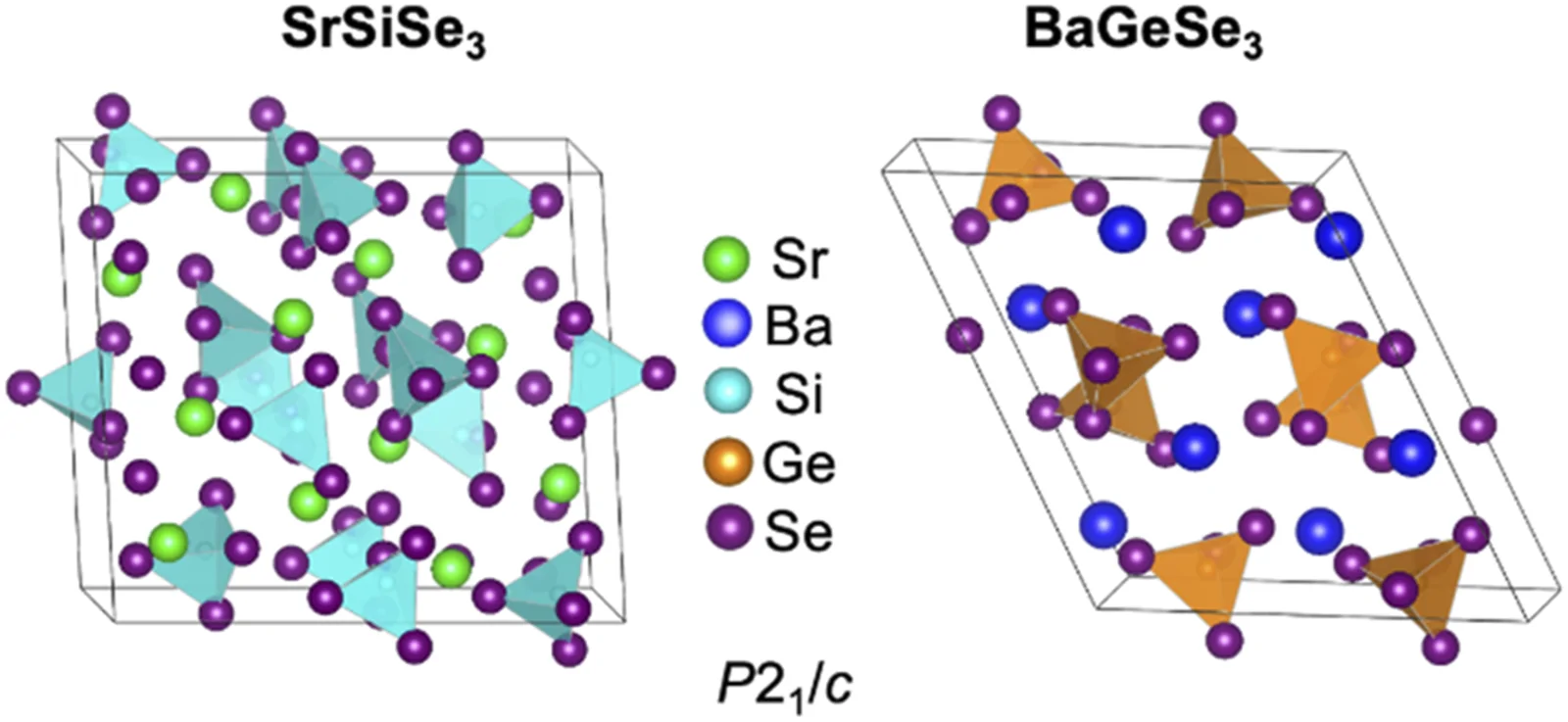

## Introduction

The growing demand for sustainable and efficient energy technologies has intensified research into advanced materials capable of addressing challenges in energy conversion and utilization. As environmental concerns and limitations of conventional energy resources become increasingly apparent, the development of materials with tailored electronic, thermal, and structural properties has emerged as a central focus in modern materials science. Applications such as in thermoelectrics,^1–4^ photovoltaics,^5–8^ and nonlinear optics,^9–12^ all rely heavily on the discovery and optimization of compounds that can deliver high performance while remaining cost-effective.

Among the broad range of candidate materials, chalcogenides have gained considerable attention due to their versatile physical properties and compositional flexibility. These compounds, formed by the combination of chalcogen elements with metals or metalloids, exhibit tunable electronic structures, high charge carrier mobility, and notable chemical stability.^13^ Such characteristics make them suitable for a variety of applications, including photovoltaics^14–17^ and thermoelectrics.^18–21^ Furthermore, their ability to form diverse crystal structures enables precise control over functional properties, particularly when engineered at reduced dimensionalities.

An additional advantage of chalcogenides lies in their relative abundance and economic viability, which align with the broader goals of sustainable materials development. Their capacity to incorporate elements with variable oxidation states—especially alkaline earth metals—facilitates the formation of structurally and chemically diverse compounds. Within this family, selenides represent an important subclass, offering distinct electronic and optical properties that have been exploited in technologies ranging from photovoltaics to optoelectronics.^13^ Well-known examples such as Cu_2_Se^22,23^ and CdSe^24,25^ highlight the technological relevance of metal selenides in both energy and electronic applications.

Ternary alkaline earth selenides such as SrSiSe_3_ and BaGeSe_3_ are of interest as emerging chalcogenide materials because the combination of an alkaline earth cation, a tetrel cation, and Se provides substantial flexibility for tuning crystal structure and electronic properties. This compositional flexibility is relevant to the development of lead-free chalcogenide semiconductors, which have attracted attention as alternatives to conventional halide perovskites (*i.e.*, CsPbCl_3_, CsPbBr_3_, CsPbI_3_),^26^ due to their chemical stability, less toxic compositions, and potentially favorable optical and electronic properties. Recent first-principles investigations of SrGeSe_3_ and BaGeSe_3_ demonstrate that these materials can possess band gaps within the technologically relevant range of approximately 0.65–2.00 eV, low exciton binding energies (6.38–73.63 meV), and carrier mobilities as high as 168 cm^2^ V^−1^ s^−1^. BaGeSe_3_ was predicted to exhibit an especially favorable combination of thermodynamic stability and high carrier mobility, highlighting its potential relevance to future photovoltaic and optoelectronic applications.^27^ Consequently, the synthesis and structural characterization of SrSiSe_3_ and BaGeSe_3_ are scientifically important not only for establishing new members of the alkaline earth selenide family, but also for defining structure–composition relationships that can guide subsequent investigation of structure–property relationships. Establishing these compounds experimentally therefore provides an essential foundation for evaluating their potential as lead-free semiconductor and optical materials.

Within this context, exploration of the A–B–Q (A = Sr–Ba; B = Si–Sn; Q = O–Te) compositional space has led to the identification of new selenides with intriguing structural features. Notably, SrSiSe_3_ was discovered as part of investigations into the Sr–Si–Se and Ba–Ge–Se systems, adding to the already existing Sr_2_SiSe_4_,^28^ Ba_7_Ge_2_Se_17_,^29^ Ba_6_Ge_2_Se_12_,^29^ Ba_2_Ge_2_Se_5_,^30^ and Ba_2_GeSe_4_,^4^ and BaGeSe_3_, as displayed in Fig. 1. For BaGeSe_3_, only space group and lattice parameters were (incorrectly) reported previously,^31^ and here we represent its full structure solution along with its electronic structure, thereby correcting both the previously experimentally and computationally determined space groups.

**Fig. 1:**
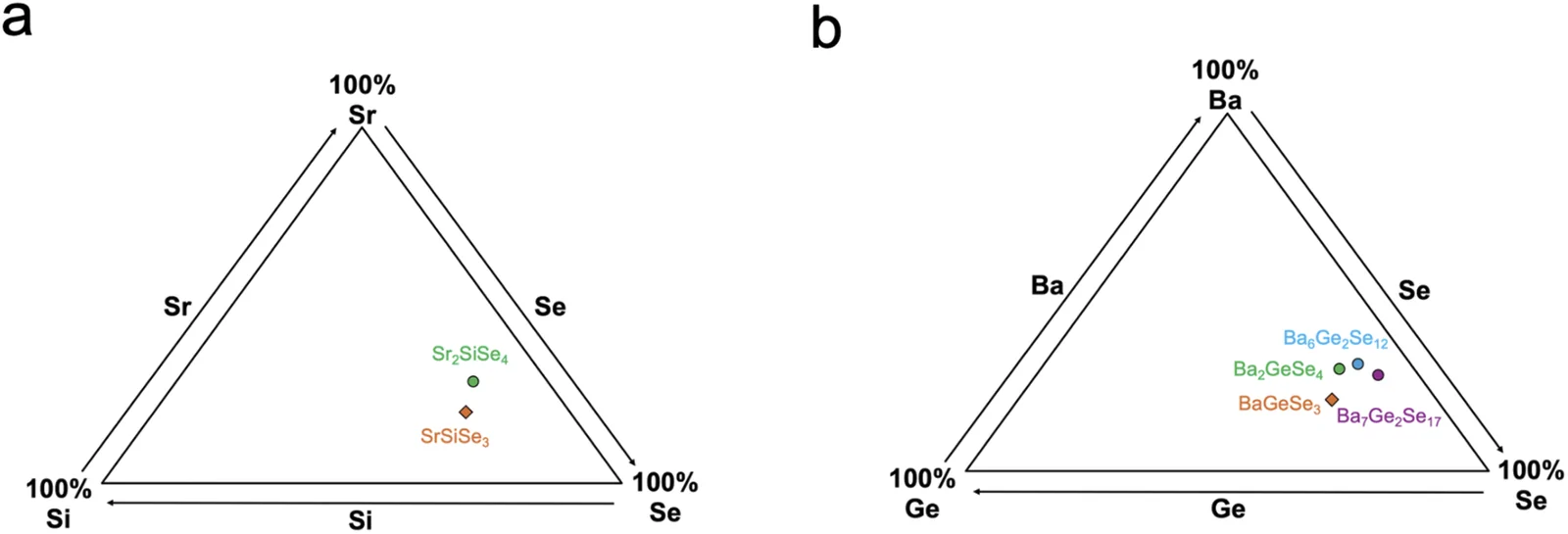
Compositional plots of similar compounds. (a) Ternary Sr–Si–Se system; (b) ternary Ba–Ge–Se system.

Despite sharing the same stoichiometric 1 : 1 : 3 ratio, these compounds exhibit distinct structural characteristics, underscoring the sensitivity of crystal chemistry to elemental substitution. In addition to the two forementioned phase systems, there are many other compounds within the ABQ_3_ family (Table 1). Additionally, SrSiS_3_ and BaSiS_3_ are reported to have been experimentally synthesized; however, there is no readily available information about their crystal structures.

**Table 1:** All known ABQ_3_ structures with experimentally determined crystal structures

Compound	Space group	Structure type	Ref
SrSiO_3_	*P* (no. 2)	BaCa_2_Si_3_O_9_	32
SrSiO_3_	*C*2 (no. 5)	CaSiO_3_	33
SrSiO_3_	*C*2/*c* (no. 15)	CaSiO_3_	34
SrSiO_3_	*P*1*c* (no. 163)	CaSiO_3_	35
SrSiO_3_	*Pmm* (no. 221)	CaTiO_3_	36
SrGeO_3_	*P* (no. 2)	BaCa_2_Si_3_O_9_	37
SrGeO_3_	*C*2/*c* (no. 15)	CaSiO_3_	38
SrGeO_3_	*P*2*m* (no. 189)	CaSiO_3_	39
SrGeO_3_	*Pmm* (no. 221)	CaTiO_3_	40
SrSnO_3_	*Pnma* (no. 62)	GdFeO_3_	41
SrSnO_3_	*Imma* (no. 74)	BaPbO_3_	42
SrSnO_3_	*I*4/*mcm* (no. 140)	SrZrO_3_	42
BaSiO_3_	*P*2_1_2_1_2_1_ (no. 19)	BaGeO_3_	43
BaSiO_3_	*Rm* (no. 166)	BaNiO_3_	44
BaSiO_3_	*P*6_3_/*mmc* (no. 194)	BaNiO_3−*x*_	44
BaGeO_3_	*C*2/*c* (no. 15)	BaSiO_3_	45
BaGeO_3_	*P*2_1_2_1_2_1_ (no. 19)	BaGeO_3_	46
BaSnO_3_	*Pmm* (no. 221)	CaTiO_3_	47
SrSnS_3_	*Pnma* (no. 62)	NH_4_CdCl_3_	48
BaGeS_3_	*P*2_1_2_1_2_1_ (no. 19)	BaGeO_3_	49
BaSnS_3_	*Pnma* (no. 62)	NH_4_CdCl_3_	48
SrSiSe_3_	*P*2_1_/*c* (no. 14)	SrGeSe_3_	This work
SrGeSe_3_	*P*2_1_/*c* (no. 14)	SrGeSe_3_	50
BaGeSe_3_	*P*2_1_/*c* (no. 14)	BaGeSe_3_	This work
BaGeSe_3_	*Pnnm* (no. 58)	?	31
BaSnSe_3_	*P*2_1_2_1_2_1_ (no. 19)	?	31

Both SrSiSe_3_ and BaGeSe_3_ crystallize in the monoclinic *P*2_1_/*c* (no. 14) space group, but adopt different structure types. Their structural frameworks provide an opportunity to examine how subtle compositional differences influence structural and electronic properties. The comparative study of SrSiSe_3_ and BaGeSe_3_ thus contributes to a deeper understanding of structure–property relationships in alkaline earth selenides and supports the ongoing development of chalcogenide-based energy materials.

## Experimental

### Synthesis and crystal growth

To synthesize SrSiSe_3_ single crystals, SrSe and SiSe_2_ were first synthesized using Sr granules (99%, Thermo Scientific), Si powder (99.9985%, Alfa Aesar), and Se powder (99.999%, Alfa Aesar). To produce SrSe, the elements were loaded into carbon-coated silica tubes and slowly heated to 873 K within 12 h and held for 6 h, then the furnace was turned off. To produce SiSe_2_, the elements were loaded into carbon-coated silica tubes and slowly heated to 973 K within 14 h and held for 6 h, then the furnace was turned off. The precursors were all ground *via* mortar and pestle, and purity was assessed using powder X-ray diffraction (PXRD). A stoichiometric 1 : 1 ratio of SrSe and SiSe_2_, respectively, was loaded into a carbon-coated silica tube which was evacuated to <2.5 × 10^−3^ mbar and slowly heated to 1073 K within 16 h and held for 72 h, then slowly cooled to 673 K over 100 h, then the furnace was turned off (Fig. S1). Transparent block-like crystals of SrSiSe_3_ were obtained with Sr_2_SiSe_4_ (ref. 28) and SiSe_2_ as a side product.

To synthesize BaGeSe_3_ single crystals, Ba pieces (99.2%, Thermo Scientific), Ge pieces (99.999%, Strem Chemicals), and Se powder (99.999%, Alfa Aesar) were used. A stoichiometric 1 : 1 : 3 ratio of Ba, Ge, and Se, respectively, was loaded into a carbon-coated silica tube which was evacuated to <2.5 × 10^−3^ mbar and slowly heated to 1073 K within 16 h and held for 72 h, then slowly cooled to 673 K over 100 h, then the furnace was turned off. Consequently, yellow block-like crystals of BaGeSe_3_ were obtained. The bulk powder was prepared using the same synthesis parameters but without slow cooling. The material appeared to be phase pure based on its PXRD diagram (Fig. S2). Additionally, synthesis of analogous compounds such as SrSiS_3_, SrGeS_3_, BaSiS_3_, SrSnSe_3_, BaSiSe_3_, BaSnSe_3_, SrSiTe_3_, SrGeTe_3_, SrSnTe_3_, BaSiTe_3_, BaGeTe_3_, and BaSnTe_3_ were attempted, but not successfully, although BaSnSe_3_ was briefly reported in 1965.^31^

### Single-crystal X-ray diffraction

Single-crystal XRD (SCXRD) experiments were performed using a Bruker Kappa Apex II CCD diffractometer equipped with Mo Kα radiation (*λ* = 0.71073 Å). A 110 × 45 × 20 µm transparent block-shaped crystal of SrSiSe_3_ was mounted, and each *ω* scan was collected for 30 s. The same process and conditions were repeated for a 64 × 60 × 20 µm yellow block-shaped crystal of BaGeSe_3_. The multi-scan method within SADABS was used for both compounds as an absorption correction method.^51^ The crystal structures were solved using the SHELXTL program.^52,53^ In both cases, the space group *P*2_1_/*c* was chosen based on the systematic extinctions that indicated the presence of the 2_1_ screw axis and the *c* glide plane. The centrosymmetric character was also confirmed in both cases by the respective |*E*^2^ – 1| values > 0.97. The excellent refinement values and featureless electron density map further support the chosen *P*2_1_/*c* as the correct space group in both cases. A twin matrix was necessary for BaGeSe_3_ to improve the w*R*_1_ and w*R*_2_ from 0.062 to 0.023 and from 0.138 to 0.040, respectively. Finally atomic positions were standardized using the program TIDY within the PLATON package.^54,55^

### Powder X-ray diffraction

PXRD patterns were collected on Malvern Panalytical Aeris diffractometer at room temperature. Diffraction patterns were collected using a Cu-Kα radiation source (*λ* = 1.5406 Å) operating at 40 kV and 15 mA over a 2*θ* range of 10–70° with a step size of 0.0054° and an exposure time of 0.36 s per step.

### SEM-EDS

Elemental analysis of the samples was obtained using SEM-EDS. The SEM-EDS analysis was performed on a Zeiss LEO 1530 instrument equipped with a Schottky-type field-emission electron gun and a backscattered electron detector, using a voltage of 20 kV. Both EDS mapping and point analyses were used to confirm elemental distribution and stoichiometry across the sample particles with an instrumental uncertainty of approximately ±5%.

### Electronic structures

The electronic structures of SrSiSe_3_ and BaGeSe_3_ were calculated using the Quantum Espresso program.^56–58^ The Perdew–Burke–Ernzerhof (PBE) functional was used as the generalized gradient approximation (GGA) for the exchange–correlation energy.^59^ A 4 × 4 × 4 grid of *k* points was used for the self-consistent field (SCF) calculations, followed by an 8 × 8 × 8 *k* point grid for the non-self-consistent field (NSCF) calculations. The criteria for energy convergence and electric charge convergence were both set to <1 × 10^−8^ Ry for the SCF and NSCF cycles. Once the convergence criteria were met, the Density Of States (DOS) were calculated.

## Results and discussion

### Crystal structure

SCXRD resulted in the charge-balanced formulas (Sr^2+^)(Si^4+^)(Se^2−^)_3_ and (Ba^2+^)(Ge^4+^)(Se^2−^)_3_. A summary of the crystallographic details is given in Table 2, the fractional coordinates and thermal displacement parameters of the symmetry independent atoms are listed in SI Tables S1 and S2, and selected interatomic distances in Tables S3 and S4. In addition, SEM-EDS data is provided in Tables S5–S8, Fig. S3 and S4 to support the forementioned crystal structure solutions.

**Table 2:** Crystal data and structure refinement for SrSiSe_3_ and BaGeSe_3_

	SrSiSe_3_	BaGeSe_3_
Molecular weight (g mol^−1^)	352.59	446.81
Crystal system	Monoclinic	Monoclinic
Space group	*P*2_1_/*c* (no. 14)	*P*2_1_/*c* (no. 14)
*a* (Å)	6.6948(2)	15.6603(7)
*b* (Å)	17.4606(4)	6.0379(3)
*c* (Å)	15.8647(3)	14.0072(6)
*β* (°)	117.0100(10)	116.3830(10)
Unit cell volume (Å^3^)	1652.23(7)	1186.50(9)
Formula units, *Z*	12	8
Temperature (K)	298(2)	297(2)
Density (g cm^−3^)	4.252	5.003
*F*(000)	1848	1520
Absorption coefficient, *µ* (mm^−1^)	29.661	29.910
Radiation wavelength (Å)	0.71073 (Mo Kα)	0.71073 (Mo Kα)
Goodness-of-fit on *F*^2^	1.029	1.136
Final *R*_1_ [*I* > 2*σ*(*I*)]^a^	0.021	0.023
Final w*R*_2_ [*I* > 2*σ*(*I*)]^a^	0.039	0.039
Largest diff. peak and hole (e Å^−3^)	0.82, −0.75	0.72, −1.05

a *σ* = standard deviation.

SrSiSe_3_ crystallizes in the centrosymmetric space group *P*2_1_/*c* (no. 14) of the monoclinic system (SrGeSe_3_ type), with unit cell parameters of *a* = 6.6948(2) Å, *b* = 17.4606(4) Å, *c* = 15.8647(3) Å, *β* = 117.0100(10)°, and *Z* = 12. There are three crystallographically unique Sr, three Si, and nine Se positions in the asymmetric unit of SrSiSe_3_, adopting the SrGeSe_3_ structure type.^50^ The twelve asymmetric units within the unit cell are displayed in Fig. 2.

**Fig. 2:**
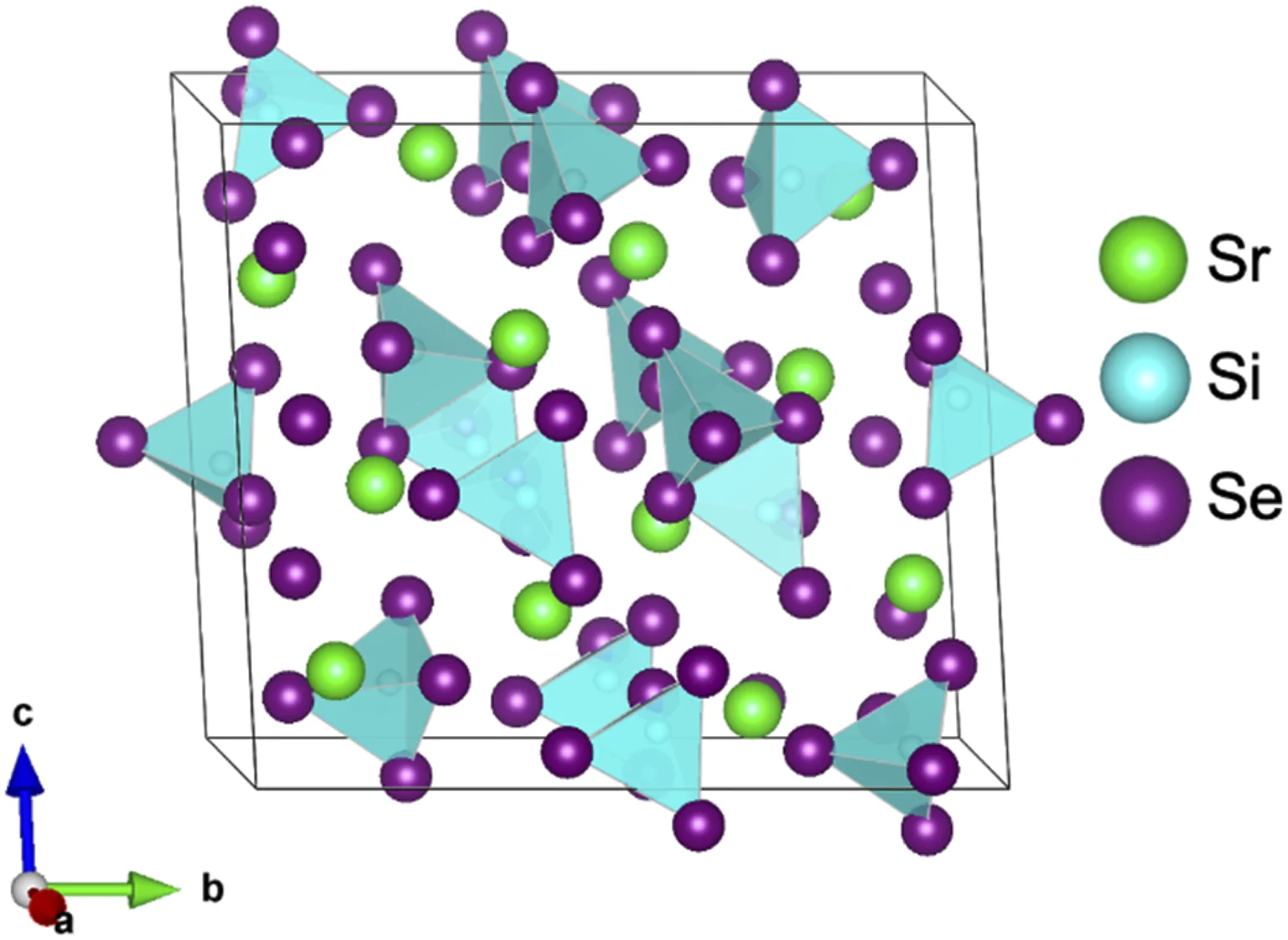
Unit cell of SrSiSe_3_.

The Sr atoms of SrSiSe_3_ have different coordination environments. Both the Sr(1) atom and the Sr(3) atom are each coordinated to eight Se atoms, thus forming Sr(1)Se_8_ and Sr(3)Se_8_ polyhedra; however, the Sr(2) atom is coordinated to seven Se atoms, forming Sr(2)Se_7_ polyhedra instead. The Si atoms are tetrahedrally coordinated to four Se atoms forming pairs of tetrahedra *via* edge-sharing. The layered structure resulting of the various polyhedra of SrSiSe_3_ is better illustrated in Fig. 3.

**Fig. 3:**
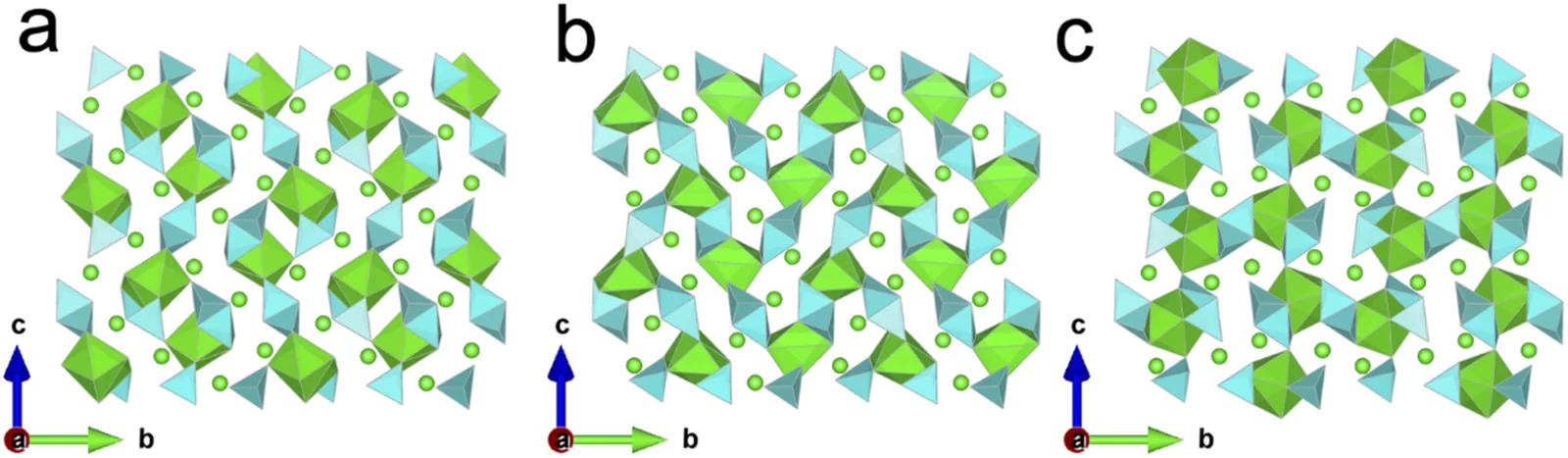
Projection of layers of SrSiSe_3_ polyhedra along the *a*-axis. (a) Sr(1)Se_8_ polyhedra and SiSe_4_ tetrahedra; (b) Sr(2)Se_7_ polyhedra and SiSe_4_ tetrahedra; (c) Sr(3)Se_8_ polyhedra and SiSe_4_ tetrahedra.

In the structure, the Sr(1)Se_8_ polyhedra are connected to the Si(1)Se_4_ and Si(2)Se_4_ tetrahedra through edge and corner-sharing Se atoms in the *b*, *c* plane, while the Sr(1)Se_8_ polyhedra are connected to the Si(3)Se_4_ through edge and corner-sharing Se atoms in the *a*, *b* plane rather than the *b*, *c* plane. The Sr(2)Se_7_ polyhedra are connected to the Si(1)Se_4_ and Si(3)Se_4_ tetrahedra through edge and corner-sharing Se atoms in the *a*, *b* plane and *b*, *c* plane, respectively; however, the Si(2)Se_4_ tetrahedra is connected to the Sr(2)Se_7_ polyhedra in a different way. The Sr(2)Se_7_–Si(2)Se_4_ connection occurs *via* both edge and corner-sharing Se atoms, not aligned along any of the axes or planes. Finally, the Sr(3)Se_8_ polyhedra are connected to the Si(1)Se_4_, Si(2)Se_4_, and Si(3)Se_4_ tetrahedra through edge and corner-sharing Se atoms in the *a*, *b* plane, *a*, *c* plane, and *b*, *c* plane, respectively. With that said, there are also Si(1)Se_4_–Si(1)Se_4_ and Si(2)Se_4_–Si(3)Se_4_ tetrahedra connected to each other through edge-sharing in the *b*, *c* plane, forming Si_2_Se_6_ units. In Fig. 3, the unique Sr(1)-centered polyhedral are shown while the other two unique Sr atoms are seen without the polyhedra to showcase their presence within the space between the layers, playing a role of balancing the charge and connecting the layers together. Due to the large Sr polyhedra, a 3D framework is formed within the crystal structure. In Fig. 4, the motifs of the SrSe_7_ and SrSe_8_ polyhedra and SiSe_4_ tetrahedra of SrSiSe_3_ are depicted to better investigate the newest member of the ABQ_3_ structural family.

**Fig. 4:**
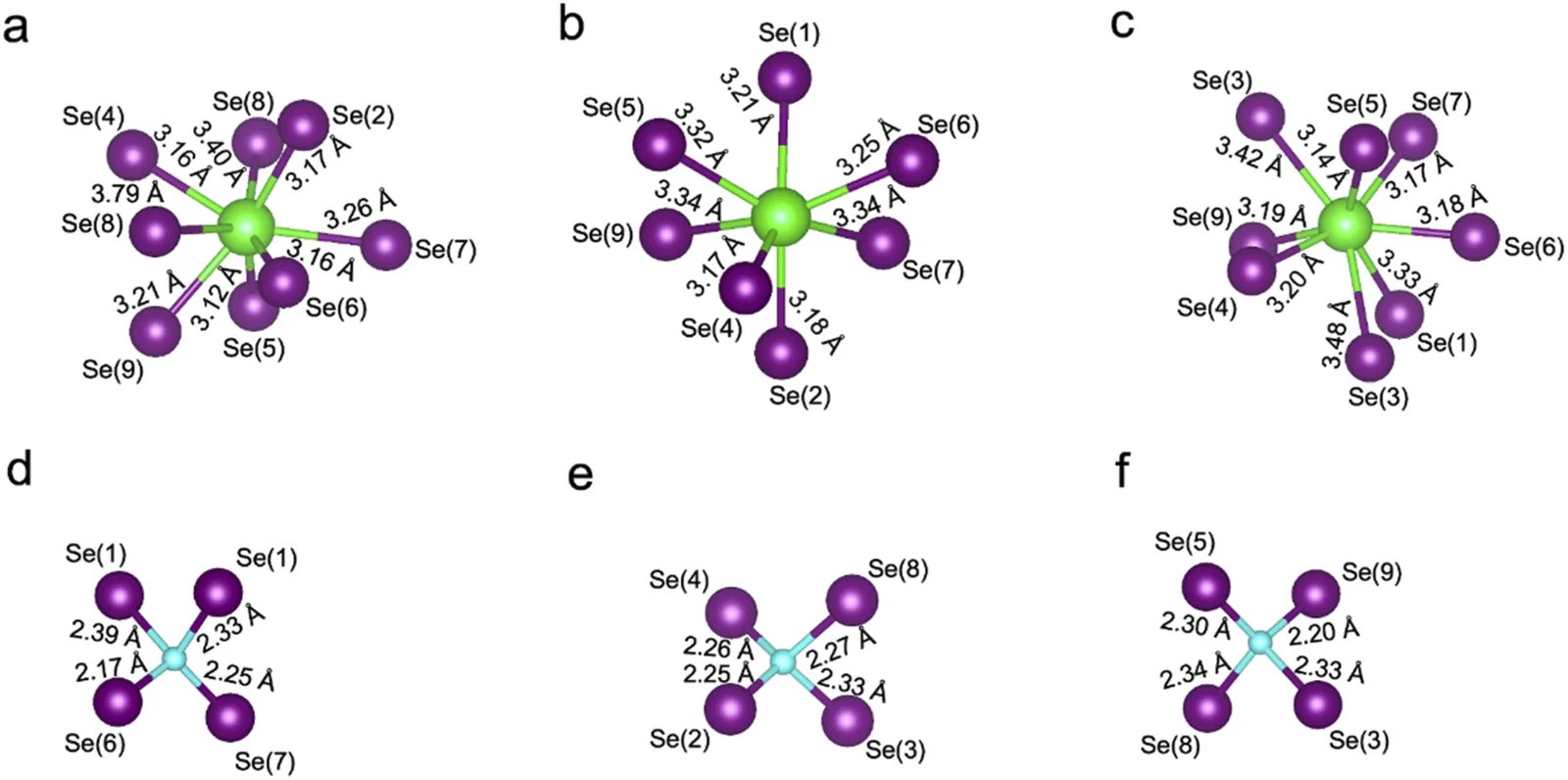
Motifs of SrSiSe_3_ polyhedra. (a) Sr(1)Se_8_ polyhedra; (b) Sr(2)Se_7_ polyhedra; (c) Sr(3)Se_8_ polyhedra; (d) Si(1)Se_4_ polyhedra; (e) Si(2)Se_4_ polyhedra; (f) Si(3)Se_4_ polyhedra.

The Sr(1)Se_8_, Sr(2)Se_7_, and Sr(3)Se_8_ polyhedra of SrSiSe_3_ all have similar Sr–Se bond lengths, where the Sr(1)Se_8_, Sr(2)Se_7_, and Sr(3)Se_8_ bond lengths range from 3.12 Å to 3.79 Å, 3.17 Å to 3.34 Å, and 3.14 Å to 3.48 Å, respectively. The Sr(1)Se_8_ and Sr(3)Se_8_ polyhedra have thus bond length ranges of 0.67 Å and 0.34 Å, whereas the Sr(2)Se_7_ polyhedra only has a range of 0.17 Å. Consequently, it is seen that the two SrSe_8_ polyhedra have more bond length variation than the SrSe_7_ polyhedra. This is likely because the seven Se atoms can form a more compact special geometry where there is lesser Se–Se repulsion, whereas this is more difficult when an eighth Se atom is added, thus why there is a greater bond length range observed with the SrSe_8_ polyhedra. The Si–Se bond lengths present in Si(1)Se_4_, Si(2)Se_4_, and Si(3)Se_4_ tetrahedra of SrSiSe_3_ are much more closely related than the Sr–Se bond lengths. The Si(1)Se_4_, Si(2)Se_4_, and Si(3)Se_4_ are 2.17 Å to 2.39 Å, 2.25 Å to 2.33 Å, and 2.20 Å to 2.34 Å, respectively. The bond length ranges of the Si(1)Se_4_, Si(2)Se_4_, and Si(3)Se_4_ tetrahedra are therefore 0.22 Å, 0.08 Å, and 0.14 Å, respectively. Much alike the Sr–Se bond lengths, the Si–Se bond lengths are reasonable based on literature.^60–65^ As expected, the SiSe_4_ tetrahedra as a collective are not as distorted as the forementioned Sr polyhedra due to less steric hindrance issues. Bond valence sum (BVS) calculations were made for Sr(1)Se_8_, Sr(2)Se_7_, Sr(3)Se_8_, Si(1)Se_4_, Si(2)Se_4_, and Si(3)Se_4_, resulting in BVS values of 1.96, 1.67, 1.94, 3.84, 3.85, and 3.74, respectively, providing additional justification that Sr and Si are present in SrSiSe_3_ in the Sr^2+^ and Si^4+^ oxidation states.^66^

Although this study features the first reported synthesis of SrSiSe_3,_ a computational study predicted the stability of various cubic perovskite phases, including SrSiSe_3_.^67^ The computational study predicted SrSiSe_3_ to be stable in form of a cubic perovskite, not adopting the *P*2_1_/*c* space group that is observed experimentally, so the stability prediction is not directly comparable in this study. With that said, SrSiSe_3_ was successfully synthesized (Fig. S1), proving the compound is stable in *P*2_1_/*c*. Unfortunately, despite attempting both binary and elemental synthesis approaches with varying temperature programs, a pure SrSiSe_3_ synthesis was not achieved as the preferred ternary phase in the compositional space is Sr_2_SiSe_4_.^28^ Consequently, this study reports SrSiSe_3_ as a minor product.

Similar to SrSiSe_3_, BaGeSe_3_ crystallizes in the centrosymmetric space group *P*2_1_/*c* (no. 14) of the monoclinic system, with unit cell parameters of *a* = 15.6603(7) Å, *b* = 6.0379(3) Å, *c* = 14.0072(6) Å, *β* = 116.3830(10)°, and *Z* = 8. There are two crystallographically unique Ba, two Ge, and six Se positions in the asymmetric unit of BaGeSe_3_, adopting its own, new structure type. The eight asymmetric units within the unit cell are displayed in Fig. 5.

**Fig. 5:**
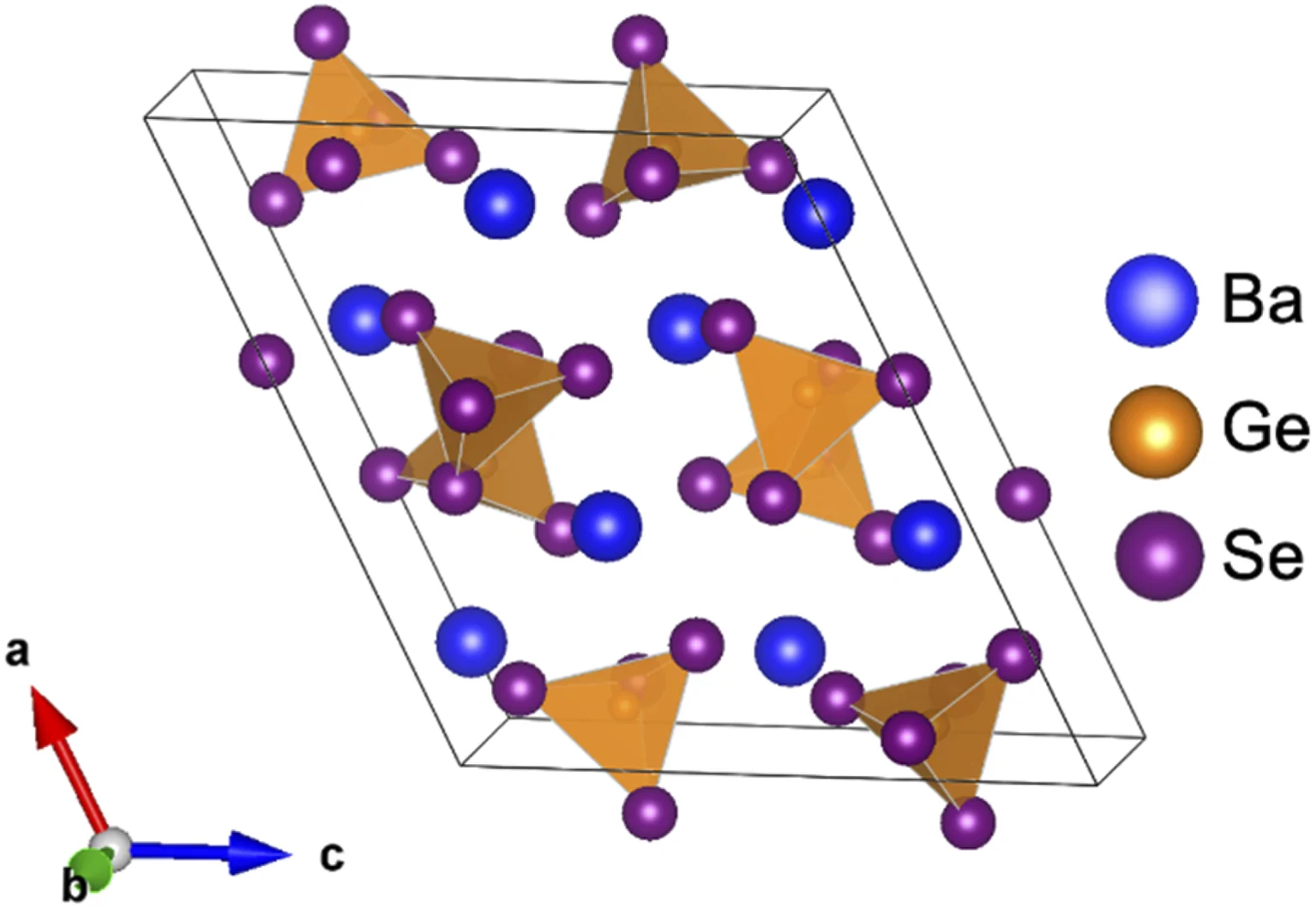
Unit cell of BaGeSe_3_.

In BaGeSe_3_, both Ba atoms are present in eight Se atoms coordination environments. The two unique Ge atoms are tetrahedrally coordinated to four Se atoms, forming GeSe_4_ units. The layered structure resulting of the various polyhedra of BaGeSe_3_ is better illustrated in Fig. 6.

**Fig. 6:**
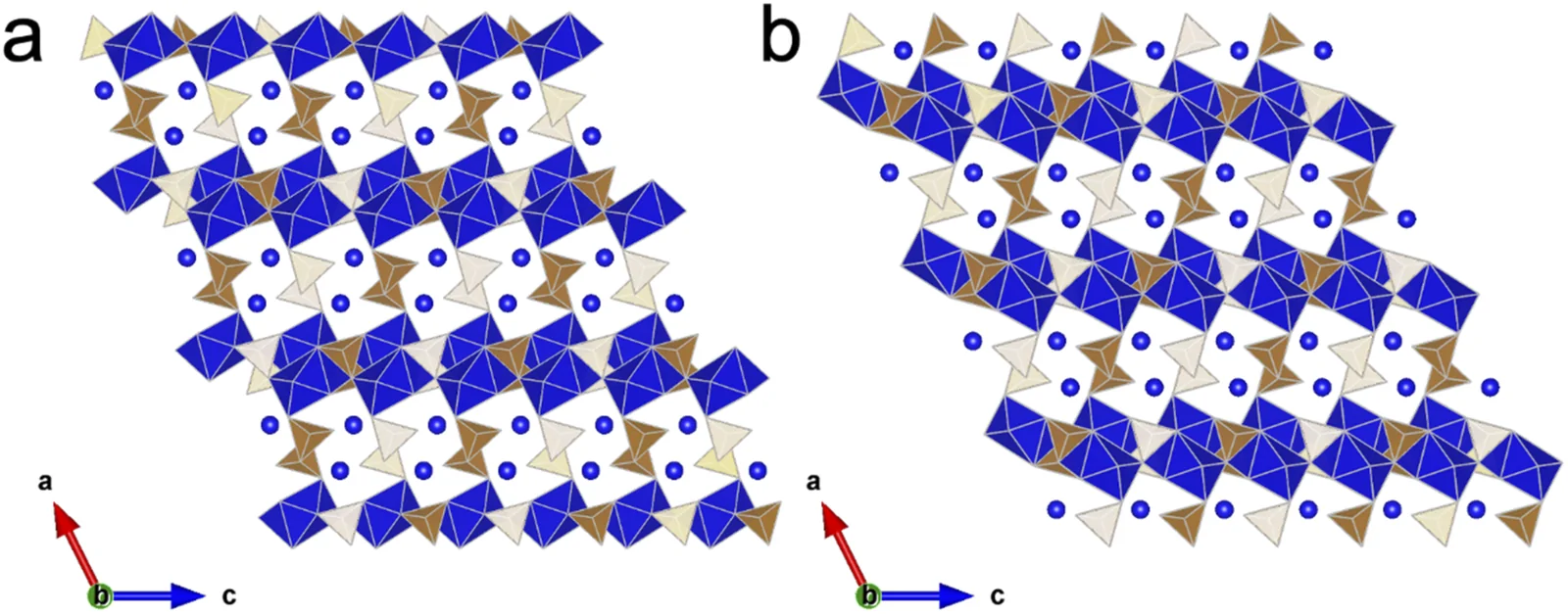
Projection of layers of BaGeSe_3_ polyhedra from the *b* axis. (a) Ba(1)Se_8_ polyhedra and GeSe_4_ tetrahedra; (b) Ba(2)Se_8_ polyhedra and GeSe_4_ tetrahedra.

The Ba(1)Se_8_ polyhedra are connected to the Ge(1)Se_4_ tetrahedra by both edge and corner-sharing Se atoms in the *b*, *c* plane, whereas the Ba(1)Se_8_ polyhedra connect to the Ge(2)Se_4_*via* only corner-sharing Se atoms in the *a*, *b* plane. The Ba(2)Se_8_ polyhedra are connected to the Ge(1)Se_4_ tetrahedra by only corner-sharing Se atoms in the *a*, *b* plane, whereas the Ba(1)Se_8_ polyhedra connects to the Ge(2)Se_4_*via* both edge and corner-sharing Se atoms in the *b*, *c* plane. Both the Ge(1)Se_4_ and Ge(2)Se_4_ tetrahedra are each connected to their own respective polyhedra types through corner-sharing Se atoms, resulting in chains forming in the *b*, *c* plane. Using the same logic as used with the Sr atoms in SrSiSe_3_, the Ba(1) and Ba(2) in BaGeSe_3_ are responsible for filling the interlayer space, providing a balanced charge, and ultimately delivering a 3D framework.

BaGeSe_3_ does not adopt any of the existing structure types of any of the known ABQ_3_ structures (Table 1). In Fig. 7, the motifs of the BaSe_8_ polyhedra and GeSe_4_ tetrahedra are shown for BaGeSe_3_.

**Fig. 7:**
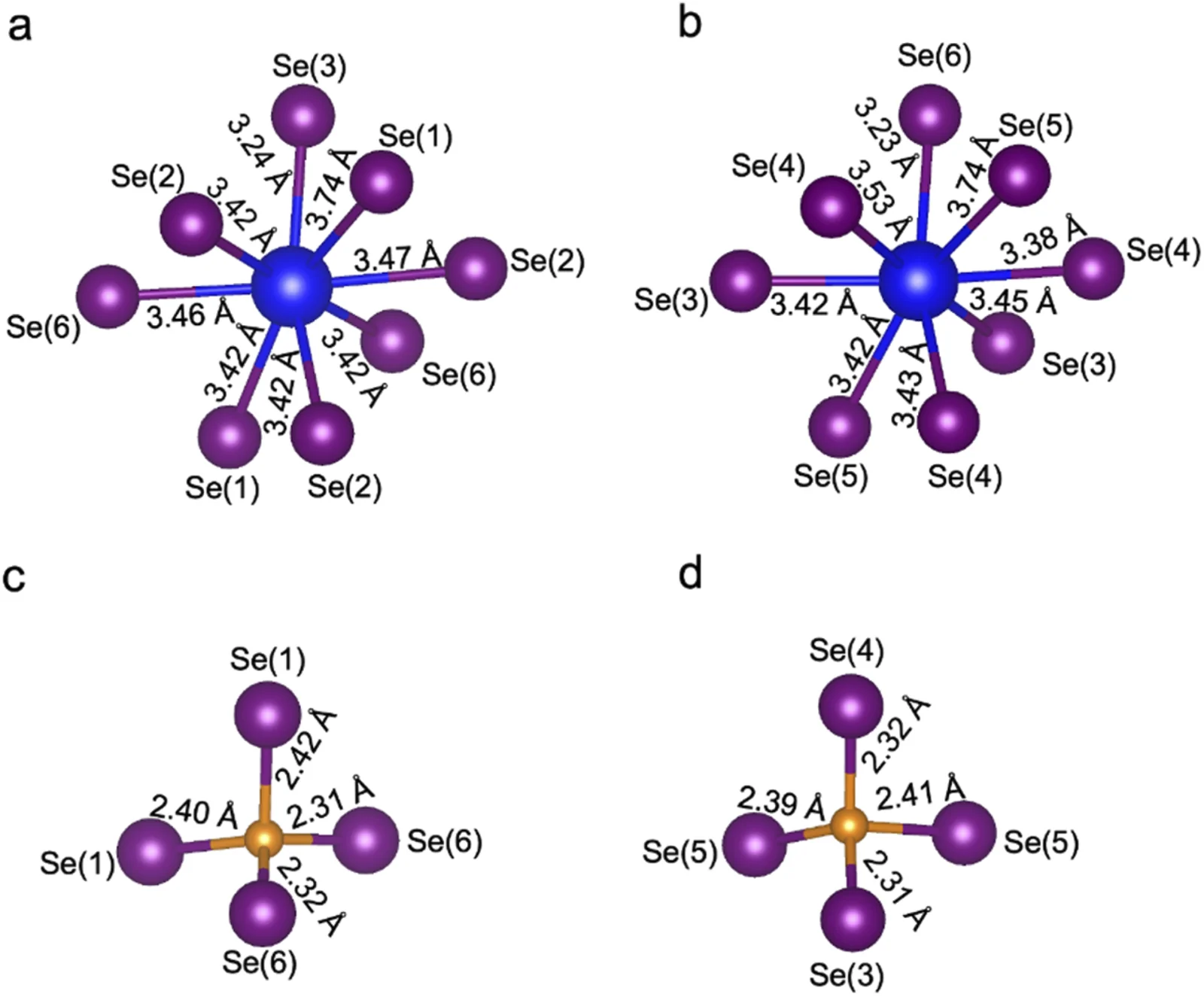
Motifs of BaGeSe_3_ polyhedra. (a) Ba(1)Se_8_ polyhedra; (b) Ba(2)Se_8_ polyhedra; (c) Ge(1)Se_4_ polyhedra; (d) Ge(2)Se_4_ polyhedra.

The Ba(1)Se_8_ and Ba(2)Se_8_ polyhedra of BaGeSe_3_ all have similar Ba–Se bond lengths; however, the Ge–Se bond lengths present in Ge(1)Se_4_ and Ge(2)Se_4_ tetrahedra of BaGeSe_3_ are much more closely related. Additionally, both the Ba–Se and Ge–Se bond lengths are within the expected range according to literature.^4,60,61,63,68^ BVS calculations^66^ were made for Ba(1)Se_8_, Ba(2)Se_8_, Ge(1)Se_4_, and Ge(2)Se_4_, resulting in BVS values of 1.82, 1.83, 3.93, and 3.93, respectively, providing additional justification that Ba and Ge are present in the 2+ and 4+ oxidation states, respectively, in BaGeSe_3_.

Table 3 demonstrates how the sizes of the A and B cations influence the B–Q coordination numbers CN and interconnection of the B–Q polyhedra. Compounds with octahedral BQ_6_ units and tetrahedral BQ_4_ units show varied connectivity depending on the specific cation sizes and packing arrangements. SrSiSe_3_ and BaGeSe_3_ both contain tetrahedral BSe_4_ units, but their different Sr/Si *versus* Ba/Ge sizes lead to different packing arrangements. In corner-sharing, two tetrahedra share one Se atom, while in edge-sharing two tetrahedra share two Se atoms, which readily explains why edge-sharing tetrahedra are easier to attain for smaller cations. The smaller Sr and Si atoms in SrSiSe_3_ allow the SiSe_4_ tetrahedra to share edges and form dimers, while the larger Ba and Ge atoms in BaGeSe_3_ favour corner-sharing GeSe_4_ tetrahedra that form chains. The same is true for SrGeSe_3_ and BaGeSe_3_ due to the size difference of Sr and Ba. Moreover, SrSnS_3_ and BaSnS_3_ are the only examples with CN = 6 for B here, because the Sn atom fits nicely into the S_6_ octahedra, in contrast to the selenides where the fit is better for Se_4_ tetrahedra rather than Se_6_ octahedra.

**Table 3:** Comparison of polyhedral interconnections in ABQ_3_ chalcogenides

ABQ_3_	Structure type	B–Q CN	Interconnection	Ref.
SrSnS_3_	NH_4_CdCl_3_	6	Edge-sharing into double chains	48
BaGeS_3_	β-BaGeO_3_	6	Corner-sharing into single chains	49
BaSnS_3_	NH_4_CdCl_3_	6	Edge-sharing into double chains	48
SrSiSe_3_	SrGeSe_3_	4	Edge-sharing into dimers	This work
SrGeSe_3_	SrGeSe_3_	4	Edge-sharing into dimers	50
BaGeSe_3_	BaGeSe_3_	4	Corner-sharing into single chains	This work

A first-principles study has predicted SrSnS_3_ and BaSnS_3_ (both NH_4_CdCl_3_ type) as high-performance thermoelectric materials.^69^ Relating structure to properties, the reason for the high thermoelectric performance could possibly be attributed to the chains of edge-sharing octahedra of SrSnS_3_ and BaSnS_3_. When corner-sharing polyhedral connections occur, there is greater room for distortion, leading to low lattice thermal conductivity; however, edge-sharing polyhedra could possibly increase the electrical transport (*i.e.* carrier mobility and thus electrical conductivity).^69,70^ This introduces an opportunity to tune structures to optimize the thermoelectric performance compounds.

Chakravorty *et al.*^27^ calculated the electronic structures of both SrGeSe_3_ and BaGeSe_3_, based on a relaxed GdFeO_3_ model (distorted perovskite). Although they reportedly found thermodynamically stable structures, those must have been only local minima in the energy landscape, as the experimentally determined structures show. Notably both materials, as discussed above, have CN = 4 for Ge, while Chakravorty *et al.* erroneously assumed CN = 6.

### Electronic structure

The electronic DOS of SrSiSe_3_ was calculated using DFT and is shown in Fig. 8. The compound is predicted to be a semiconductor with a calculated band gap of 2.15 eV, which is to be expected by its transparent crystals, considering that DFT typically underestimates the band gap. The valence band is mainly comprised of the 4p orbitals of the Se atoms, although from −4.5 to −2.5 eV there is also a moderate contribution from the Si 3p orbital. Contrastingly, the conduction band has similar contribution from the Si 3p and Se 4p orbitals on the moderate side, whereas the Sr 4d orbitals displayed lower contribution.

**Fig. 8:**
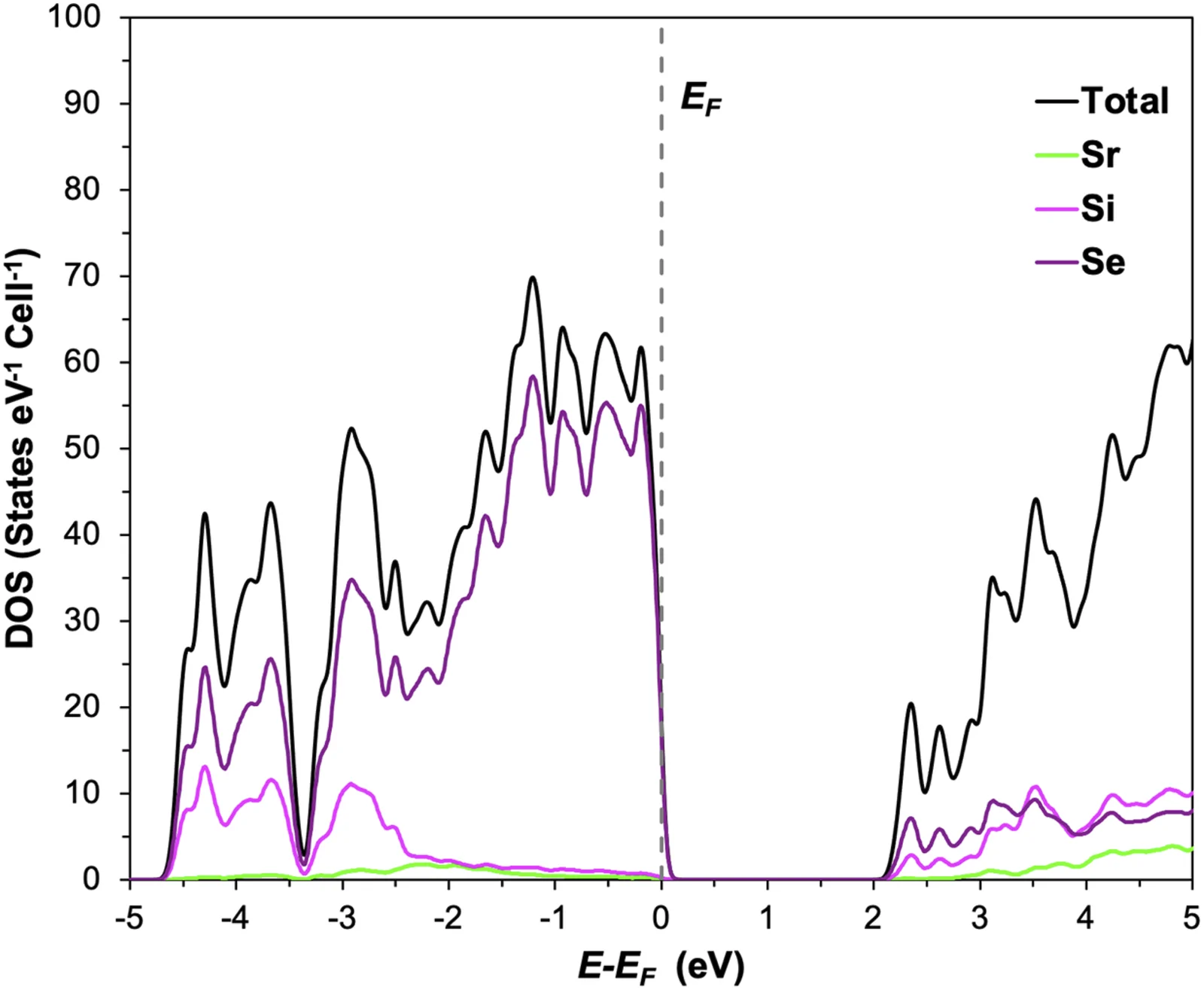
Calculated density of states of SrSiSe_3_.

The electronic DOS of BaGeSe_3_ was calculated using DFT and is shown in Fig. 9. The compound is predicted to be a semiconductor with a calculated band gap of 1.65 eV, which is to be expected by its yellow crystals. From −4.5 eV to −2.0 eV, the valence band has moderate contribution from the 4p orbitals of the Ge atoms, whereas the entirety of the valence band has major and minor contribution from the Se 4p and Ba 6s orbitals, respectively. Contrastingly, the conduction band has high contribution from the Se 4p orbitals from 1.5 to 3.0 eV, and low contribution from the Ge 4p and Ba 6s orbitals; however, beyond 3.0 eV of the conduction band displays low to moderate contribution from the Ba 6s, Ge 4p, and Se 4p orbitals.

**Fig. 9:**
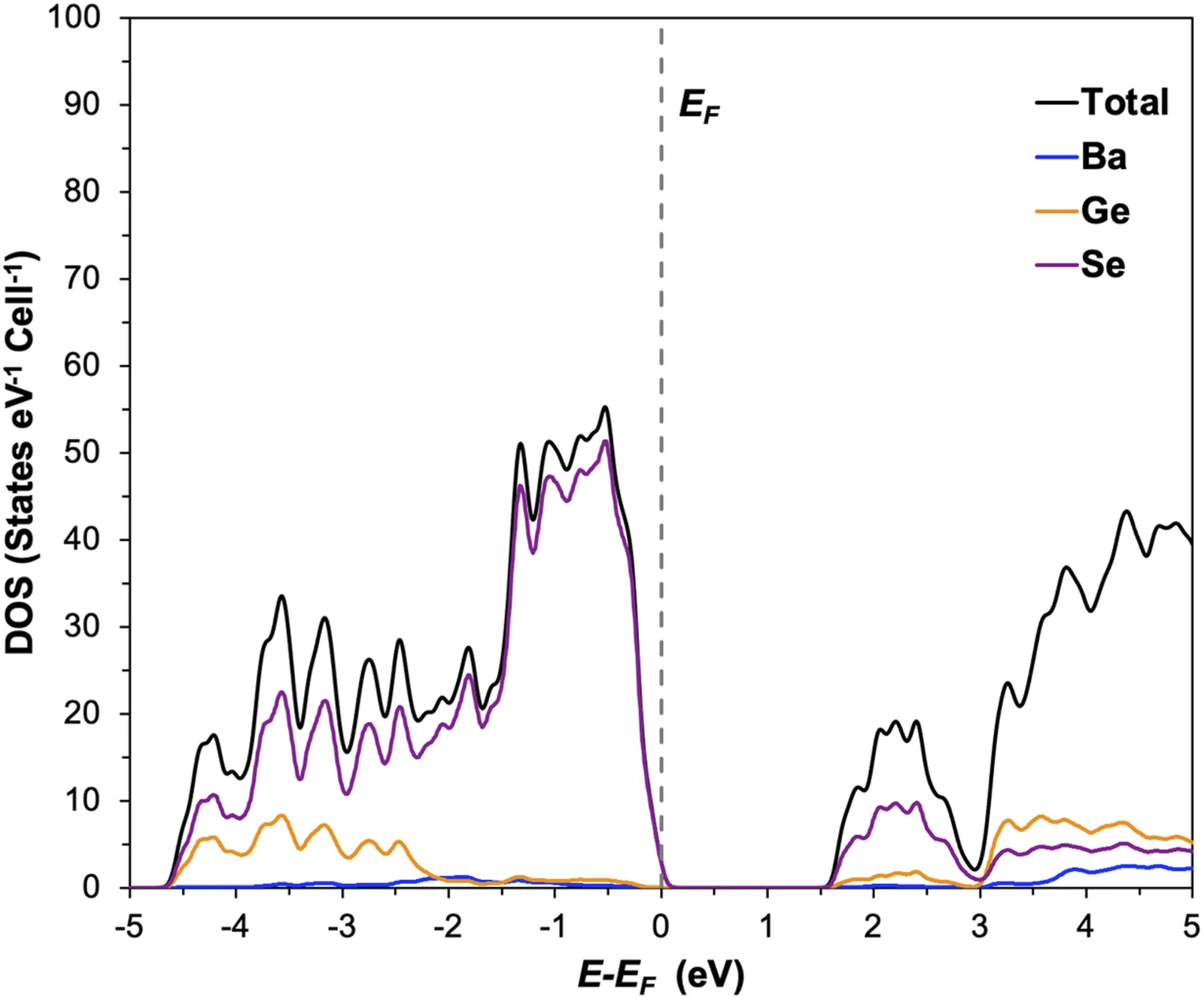
Calculated density of states of BaGeSe_3_.

## Conclusions

This study reports the experimental crystal structures of the ternary selenides SrSiSe_3_ and BaGeSe_3_. The contrasting structural arrangements within the same ABQ_3_ stoichiometry highlight the substantial structural diversity that can arise from relatively simple chemical compositions. Comparison of the experimental structures with previously calculated and reported ABQ_3_ phases demonstrates that computationally used structures do not always correspond to the experimentally accessible phases, emphasizing the importance of evaluating computed crystal structures. DFT calculations further show that SrSiSe_3_ (*E*_g_ = 2.15 eV) and BaGeSe_3_ (*E*_g_ = 1.65 eV) are semiconductors within the visible-energy range. Together, these results expand the structural landscape of the ABQ_3_ ternary selenide family and provide experimentally validated structures that can serve as benchmarks for future computational predictions and investigations of structure–property relationships.

## Author contributions

Tristan Licskai: conceptualization, data curation, formal analysis, investigation, visualization, writing – final draft; Abdeljalil Assoud: data curation, formal analysis; Ehsan Niknam: data curation, formal analysis; Holger Kleinke: conceptualization, funding acquisition, supervision, writing – review and editing.

## Conflicts of interest

There are no conflicts to declare.

## Supplementary Material

RA-016-D6RA08053G-s001

RA-016-D6RA08053G-s002

## Data Availability

The data supporting this article have been included as part of the supplementary information (SI). Supplementary information: powder X-ray diffraction patterns, atomic coordinates and equivalent isotropic displacement parameters, interatomic distances, and SEM-EDS data. See DOI: https://doi.org/10.1039/d6ra08053g. CCDC 2583081 (BaGeSe_3_) and 2583082 (SrSiSe_3_) contain the supplementary crystallographic data for this paper.^71*a*,*b*^
