# Supplementary material for: Crystal structures and electronic structures of SrSiSe3 and BaGeSe3
Source: RSC Adv. 2026 Sep 29;16(45):58516–24. doi: 10.1039/d6ra08053g (PMC13621485; doi:10.1039/d6ra08053g)
Supplement: Supplementary file 1 [file RA-016-D6RA08053G-s001.pdf]

Supplementary Information (SI) for RSC Advances.
This journal is © The Royal Society of Chemistry 2026

RSC Advances

Crystal Structures and Electronic Structures of $SrSiSe_3$ and $BaGeSe_3$

Tristan Licskai, ORCID: 0009-0002-1366-7587

Abdeljalil Assoud, ORCID: 0000-0001-6401-8478

Ehsan Niknam, ORCID: 0000-0003-1020-9046

Holger Kleinke*, ORCID: 0000-0002-6777-6140

Department of Chemistry and Waterloo Institute for Nanotechnology, University of Waterloo, Waterloo, Ontario N2L 3G1, Canada.

***Correspondence to:** Prof. Holger Kleinke, Department of Chemistry, University of Waterloo, 200 University Ave W., Waterloo, Ontario N2L 3G1, Canada. E-mail: kleinke@uwaterloo.ca.

Supporting Information

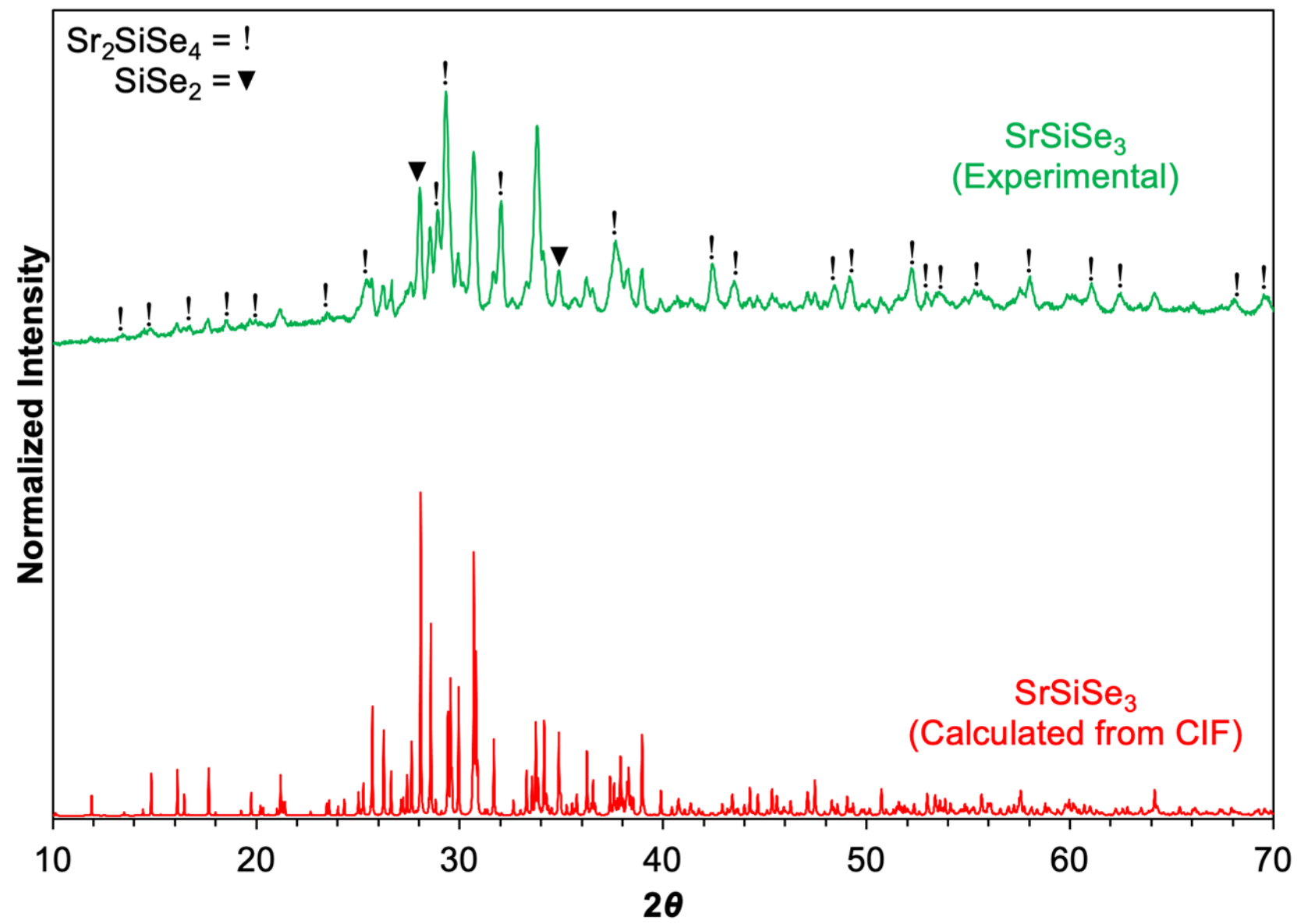

Fig. S1 Experimental and calculated PXRD patterns of $SrSiSe_3$. The CIF file was created from our single crystal structure data.

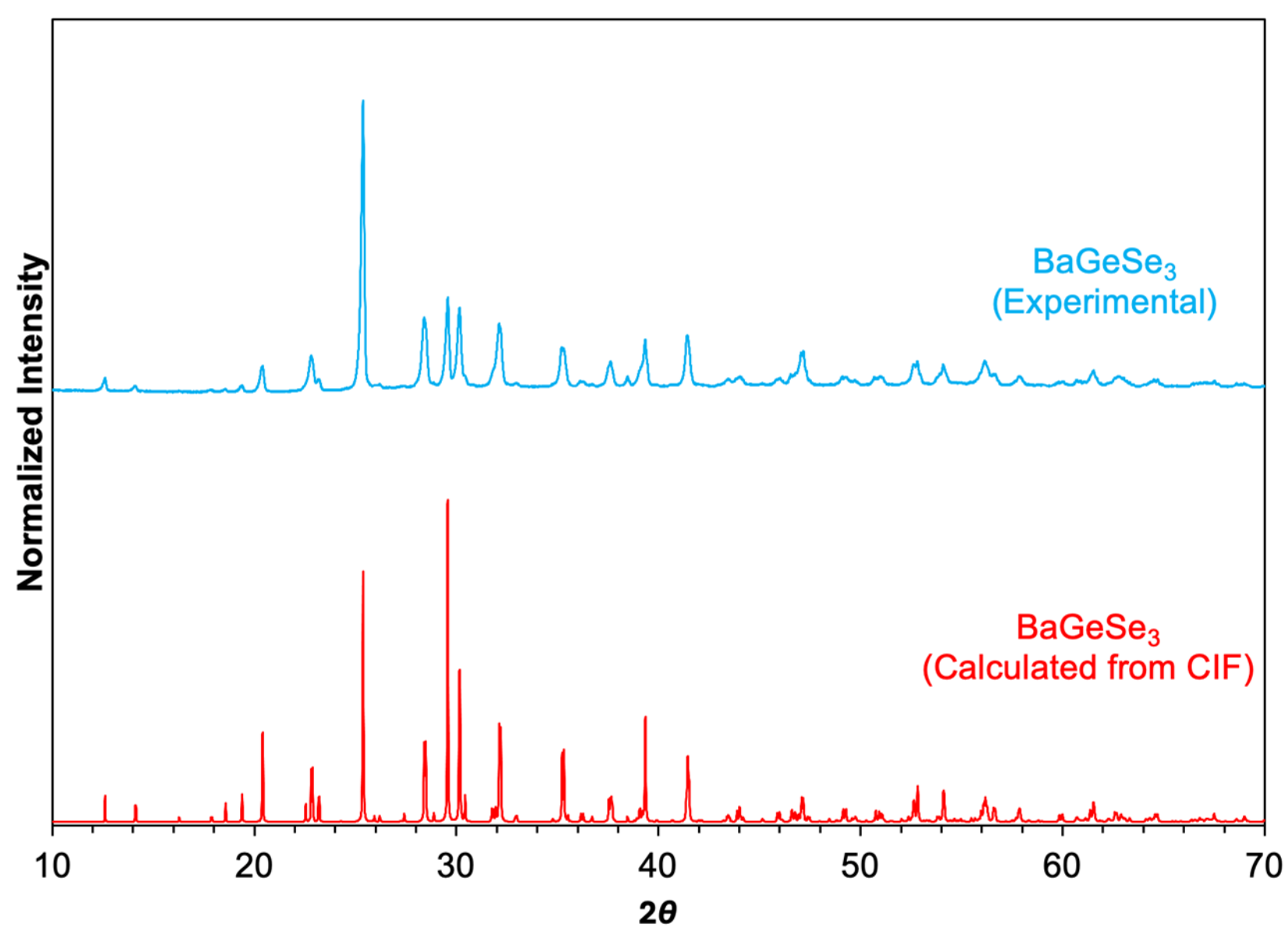

Fig. S2 Experimental and calculated PXRD patterns of $BaGeSe_3$. The CIF file was created from our single crystal structure data. The differences in peak intensities are caused by the texture effect.

Table S1 Atomic coordinates and equivalent isotropic displacement parameters for $SrSiSe_3$.

Atom	Wyckoff Position	*x*	*y*	*z*	*U* (Å^2)
Sr(1)	4 *e*	0.03872(5)	0.61591(2)	0.32581(2)	0.01548(7)
Sr(2)	4 *e*	0.53856(5)	0.42136(2)	0.23256(2)	0.02062(7)
Sr(3)	4 *e*	0.61928(5)	0.20358(2)	0.42845(2)	0.01638(7)
Si(1)	4 *e*	0.02901(13)	0.03842(5)	0.41507(6)	0.01032(16)
Si(2)	4 *e*	0.05901(13)	0.37608(5)	0.44268(6)	0.01249(17)
Si(3)	4 *e*	0.37841(13)	0.22310(5)	0.11221(6)	0.01151(17)
Se(1)	4 *e*	0.04407(5)	0.41244(2)	0.05812(2)	0.01211(7)
Se(2)	4 *e*	0.06776(5)	0.43503(2)	0.31861(2)	0.01695(7)
Se(3)	4 *e*	0.15187(5)	0.24688(2)	0.45398(2)	0.01391(7)
Se(4)	4 *e*	0.26907(5)	0.61705(2)	0.54941(2)	0.01640(7)
Se(5)	4 *e*	0.27406(5)	0.25896(2)	0.22608(2)	0.01373(7)
Se(6)	4 *e*	0.27717(5)	0.58377(2)	0.19757(2)	0.01273(7)
Se(7)	4 *e*	0.32012(5)	0.05416(2)	0.38226(2)	0.01385(7)
Se(8)	4 *e*	0.33660(5)	0.09098(2)	0.08713(2)	0.01376(7)
Se(9)	4 *e*	0.70059(5)	0.27608(2)	0.14120(2)	0.01526(7)

Table S2 Atomic coordinates and equivalent isotropic displacement parameters for $BaGeSe_3$.

Atom	Wyckoff Position	*x*	*y*	*z*	*U* (Å^2)
Ba(1)	4 *e*	0.13719(3)	0.25522(6)	0.02851(3)	0.02198(8)
Ba(2)	4 *e*	0.63661(3)	0.26119(6)	0.10829(3)	0.02342(8)
Ge(1)	4 *e*	0.07442(4)	0.65028(9)	0.26817(4)	0.01233(12)
Ge(2)	4 *e*	0.42539(4)	0.35445(9)	0.19501(4)	0.01230(12)
Se(1)	4 *e*	0.08388(4)	0.25141(8)	0.26339(4)	0.01555(11)
Se(2)	4 *e*	0.10411(4)	0.74957(8)	0.12573(4)	0.01907(12)
Se(3)	4 *e*	0.31861(4)	0.26453(9)	0.26164(4)	0.01642(11)
Se(4)	4 *e*	0.39484(4)	0.25349(8)	0.02328(4)	0.01948(12)
Se(5)	4 *e*	0.58364(4)	0.25293(9)	0.32010(4)	0.01578(11)
Se(6)	4 *e*	0.81951(4)	0.24042(9)	0.05948(4)	0.01661(11)

Table S3 Interatomic distances for $SrSiSe_3$.

Bond Pair	Bond Length (Å)
Sr(1)–Se(2)	3.1694(5)
Sr(1)–Se(4)	3.1612(4)
Sr(1)–Se(5)	3.1202(4)
Sr(1)–Se(6)	3.1548(4)
Sr(1)–Se(7)	3.2599(4)
Sr(1)–Se(8)	3.4004(4)
Sr(1)–Se(8)	3.7869(4)
Sr(1)–Se(9)	3.2113(5)
Sr(2)–Se(1)	3.2126(4)
Sr(2)–Se(2)	3.1748(4)
Sr(2)–Se(4)	3.1665(5)
Sr(2)–Se(5)	3.3197(5)
Sr(2)–Se(6)	3.2450(5)
Sr(2)–Se(7)	3.3400(5)
Sr(2)–Se(9)	3.3376(5)
Sr(3)–Se(1)	3.3332(4)
Sr(3)–Se(3)	3.4174(4)
Sr(3)–Se(3)	3.4838(4)
Sr(3)–Se(4)	3.2019(5)
Sr(3)–Se(5)	3.1375(4)
Sr(3)–Se(6)	3.1801(4)
Sr(3)–Se(7)	3.1658(4)
Sr(3)–Se(9)	3.1853(4)
Si(1)–Se(1)	2.3341(9)
Si(1)–Se(1)	2.3851(9)
Si(1)–Se(6)	2.1668(8)
Si(1)–Se(7)	2.2509(8)
Si(2)–Se(2)	2.2453(9)
Si(2)–Se(3)	2.3252(9)
Si(2)–Se(4)	2.2594(9)
Si(2)–Se(8)	2.2733(8)
Si(3)–Se(3)	2.3278(9)
Si(3)–Se(5)	2.2999(9)
Si(3)–Se(8)	2.3359(9)
Si(3)–Se(9)	2.1952(8)

Table S4 Interatomic distances for $BaGeSe_3$.

Bond Pair	Bond Length (Å)
Ba(1)–Se(1)	3.4254(6)
Ba(1)–Se(1)	3.7367(6)
Ba(1)–Se(2)	3.4146(6)
Ba(1)–Se(2)	3.4194(7)
Ba(1)–Se(2)	3.4743(6)
Ba(1)–Se(3)	3.2393(6)
Ba(1)–Se(6)	3.4172(6)
Ba(1)–Se(6)	3.4635(6)
Ba(2)–Se(3)	3.4159(6)
Ba(2)–Se(3)	3.4514(6)
Ba(2)–Se(4)	3.3791(6)
Ba(2)–Se(4)	3.4276(7)
Ba(2)–Se(4)	3.5340(6)
Ba(2)–Se(5)	3.4177(6)
Ba(2)–Se(5)	3.7435(6)
Ba(2)–Se(6)	3.2290(7)
Ge(1)–Se(1)	2.3958(7)
Ge(1)–Se(1)	2.4157(7)
Ge(1)–Se(2)	2.3197(7)
Ge(1)–Se(6)	2.3063(7)
Ge(2)–Se(3)	2.3128(7)
Ge(2)–Se(4)	2.3160(7)
Ge(2)–Se(5)	2.3935(7)
Ge(2)–Se(5)	2.4138(7)

Table S5 Area SEM–EDS map data for $SrSiSe_3$.

Shell	Experimental at.-%	Error %	Theoretical at.-%
Sr-L	20.8	±1.0	20.0
Si-K	22.3	±1.0	20.0
Se-L	56.9	±3.0	60.0

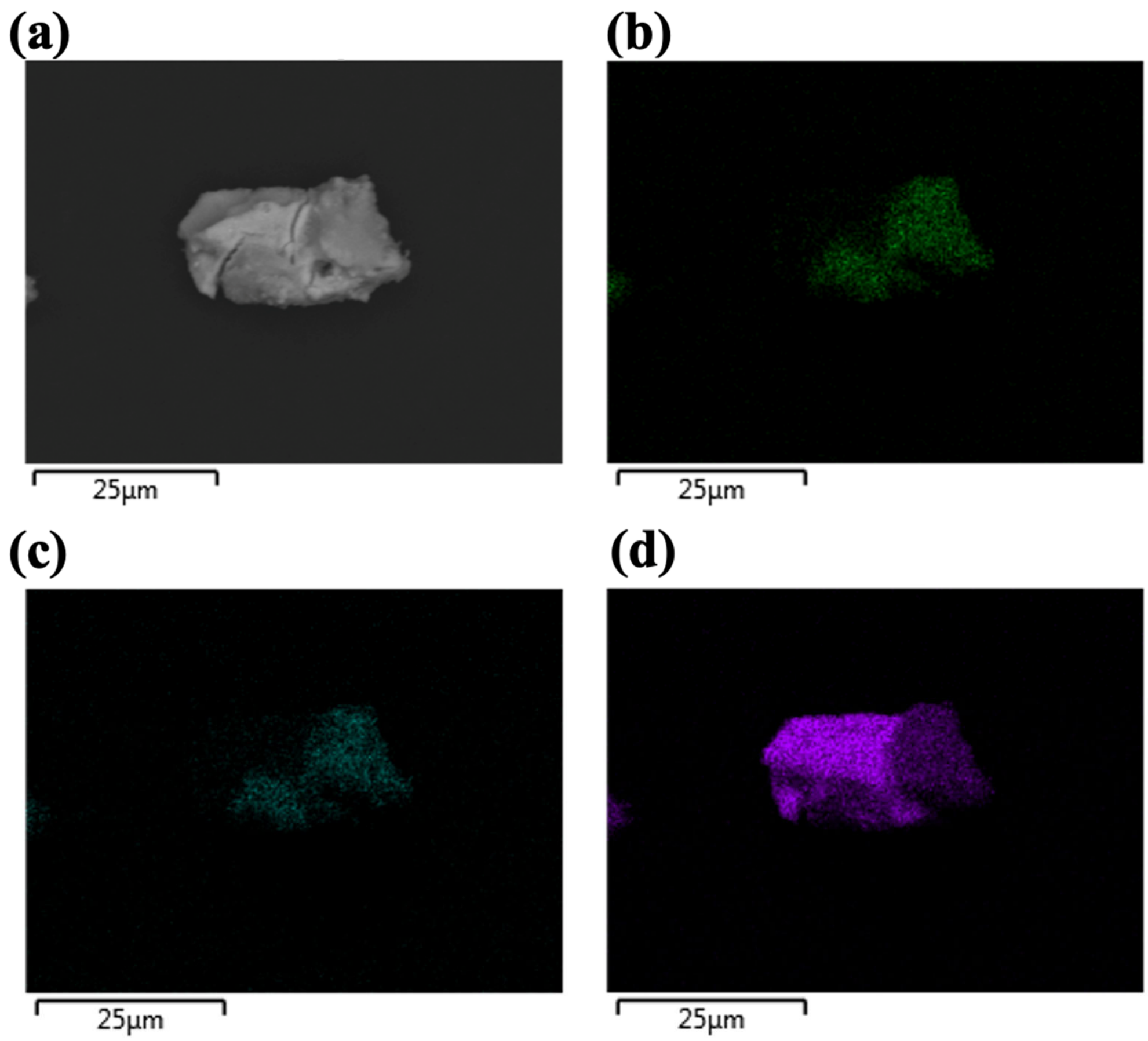

Figure S3 SEM–EDS maps for $SrSiSe_3$. (**a**) SEM image; (**b**) EDS map of Sr; (**c**) EDS map of Si; (**d**) EDS map of Se.

Table S6 Point EDS data for $SrSiSe_3$.

Shell	Point 1 at.-%	Point 2 at.-%	Point 3 at.-%	Point 4 at.-%	Point 5 at.-%	Ave. at.-%	STD	Theoretical at.-%
Sr-L	23.7	27.4	26.6	24.4	26.1	25.6	±1.5	20.0
Si-K	19.3	19.4	17.5	15.8	21.5	18.7	±2.2	20.0
Se-L	57	53.2	55.9	59.8	52.4	55.7	±3.0	60.0

Table S7 Area SEM–EDS data for $BaGeSe_3$.

Shell	Experimental at.-%	Error %	Theoretical at.-%
Ba-L	17.7	±1.0	20
Ge-K	19.8	±1.0	20
Se-L	62.5	±3.0	60

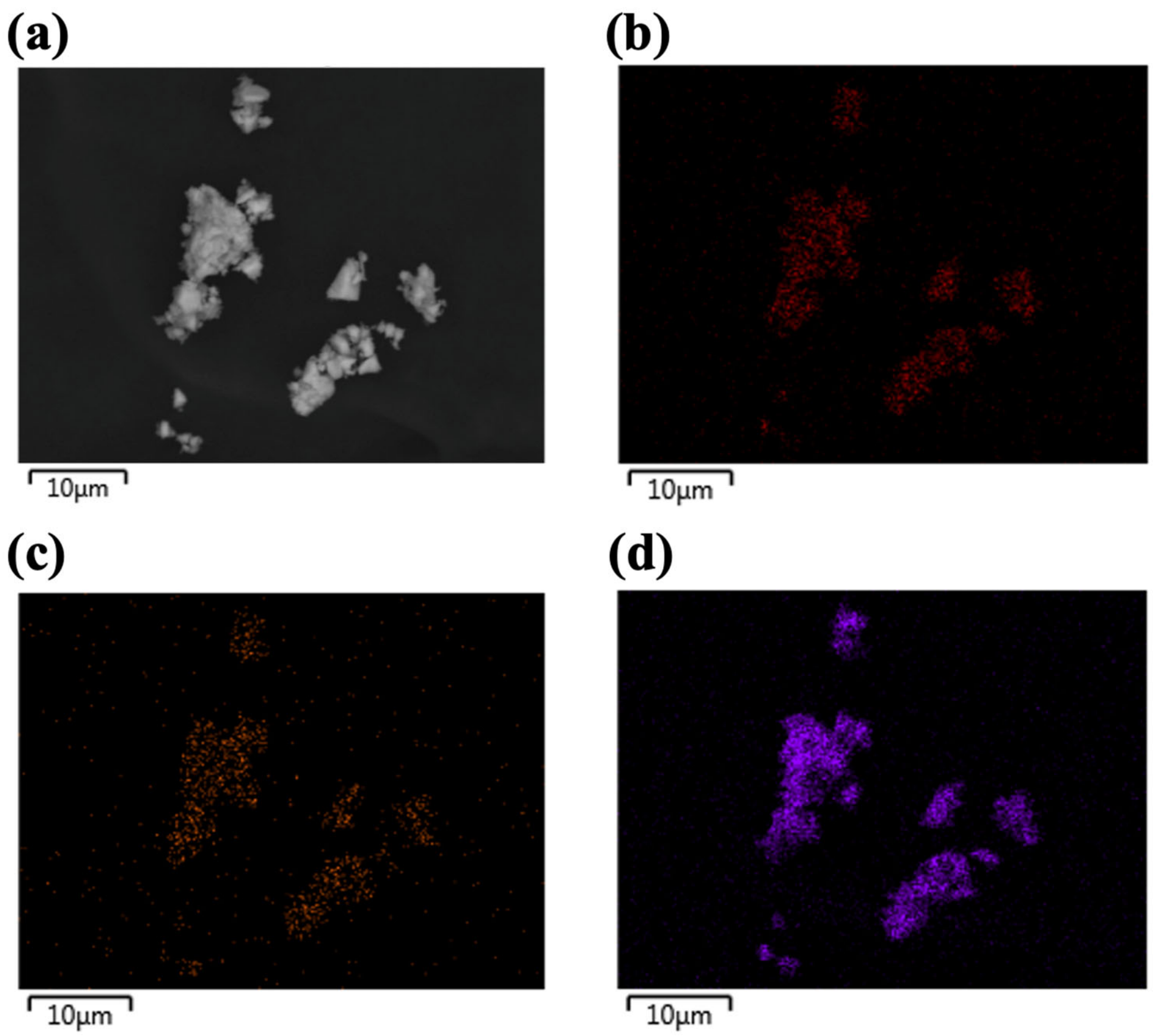

Figure S4 SEM–EDS maps for $BaGeSe_3$. (**a**) SEM image; (**b**) EDS map of Ba; (**c**) EDS map of Ge; (**d**) EDS map of Se.

Table S8 Point EDS data for $BaGeSe_3$.

Shell	Point 1 at.-%	Point 2 at.-%	Point 3 at.-%	Point 4 at.-%	Point 5 at.-%	Ave. at.-%	STD	Theoretical at.-%
Ba-L	19.2	19.3	20.3	19.0	18.0	19.2	±0.8	20
Ge-K	20.9	21.6	21.6	18.9	19.3	20.5	±1.3	20
Se-L	59.9	59.1	58.1	62.2	62.7	60.4	±2.0	60
